# A Case of Sarcoidosis of the Central Nervous System and Orbita

**DOI:** 10.1155/2015/403459

**Published:** 2015-02-25

**Authors:** Metin Mercan, Aslı Akyol, Yahya Karaman, Hayrunnisa Bolay

**Affiliations:** Department of Neurology, Gazi University Faculty of Medicine, 06410 Ankara, Turkey

## Abstract

Sarcoidosis is a multisystemic disease characterized by granulomatous inflammation. Lung or lymph node involvement is common. We present a rare case of sarcoidosis that began with orbital involvement, and a month later, due to insufficient treatment, it involved the central nervous system. A 49-year-old female patient began suffering from swelling in her right eye, redness, ptosis, and limited eye movements two months ago. Gadolinium-enhanced orbital magnetic resonance imaging showed thickening of the lacrimal gland and the right medial rectus muscle. After three weeks of local antibiotic and steroid treatments, her symptoms were resolved. One month ago, the patient reported sudden weakness in her right arm and leg. After laboratory tests and imaging studies, the patient was diagnosed with probable neurosarcoidosis using the Zajicek criteria and treated with prednisone (1 mg/kg/day). Although sarcoidosis frequently presents with lung and lymph node involvement, it is rarely accompanied by orbital involvement. Patients with orbital symptoms may receive a late diagnosis and insufficient central nervous system treatment. Involvement of the central nervous system in sarcoidosis leads to high morbidity and mortality rates. Therefore, early diagnosis and treatment are very important.

## 1. Introduction

Sarcoidosis is a chronic multisystemic disease with progressive formation of noncaseating granulomas that can develop in organs, including the lungs, lymph nodes, eyes, and skin [1]. While symptoms may appear at any age, peak occurrence is between 30 and 40 years of age [2]. Central nervous system involvement (neurosarcoidosis) occurs in 5–15% of cases. Patients with neurosarcoidosis present with neurologic symptoms, including headaches, visual impairment, diplopia, ataxia, motor deficits, seizures, and cognitive decline [3, 4]. Ocular involvement typically presents as uveitis and, rarely, as orbital involvement affecting the lacrimal gland and extraocular muscles. Isolated sarcoidosis unaccompanied by systemic involvement is rare and difficult to diagnosis [5, 6]. 

## 2. Case Presentation

**Figure 1:**
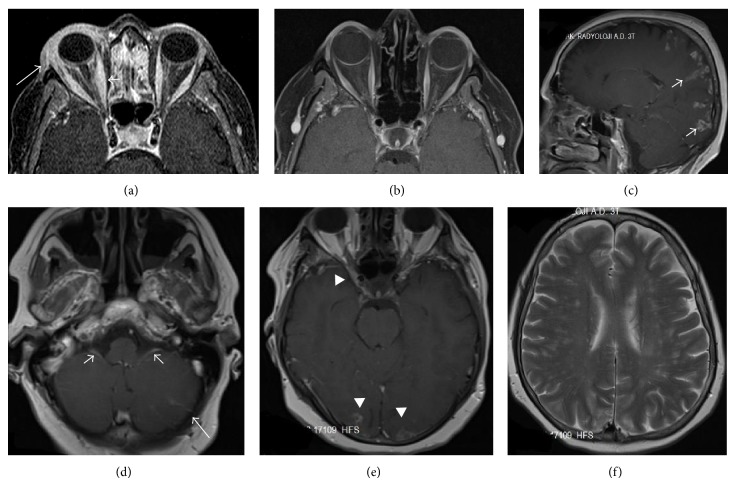
Thickening of the medial rectus muscle (short arrow; right diameter 9.5 mm; left diameter 5.8 mm) with gadolinium enhancement of the right lacrimal gland (long arrow) and periorbital fatty tissue in the axial fat-suppressed postcontrast T1-weighted sequences (a). The lacrimal gland and the medial rectus muscle appear normal in the axial fat-suppressed postcontrast T1-weighted sequences (b). Pial-gyral nodular gadolinium-enhanced lesions (short arrow) are detected in the bilateral occipital and parietal areas in the sagittal postcontrast T1-weighted sequences (c). Pial gadolinium enhancement (long arrow) and diffuse enhancement of the 9th and 10th cranial nerves (short arrow) are observed in both cerebellar hemispheres in the axial postcontrast T1-weighted sequences (d). Pial-gyral nodular gadolinium enhancement was also observed to be adjacent to the right optic foramen and the bilateral occipital area in axial postcontrast T1-weighted sequences (arrowhead) (e). In axial T2-weighted sequences, hyperintense multiple nodular lesions were detected in the bilateral periventricular white matter (f).

For years, a 49-year-old female suffered from sudden eruptions of erythematous that recovered without treatment. Two months ago, magnetic resonance imaging (MRI) was performed due to swelling, redness, ptosis, and restriction of movement in the patient's right eye. The MRI revealed thickening of the right lacrimal gland and the medial rectus muscle and the contrast agent was retained in the lacrimal gland (Figure 1(a)). The patient's symptoms receded after three weeks of treatment with local steroids and antibiotics. One month ago, the patient suddenly developed muscle weakness in the right arm and leg. She improved after four days of treatment with 1,000 mg/day of methylprednisolone.

After treatment, the patient continued to suffer and returned to our hospital to have her complaints addressed. There was nothing notable in the personal or family medical histories. The physical examination revealed no positive findings. During the neurological examination, we identified a bilateral reduction of the gag reflex, muscular weakness in the upper and lower right extremities (4/5), increased deep tendon reflexes, and the Babinski sign. The brain MRI revealed pial-gyral gadolinium enhancement in the cranium base, the 9th and 10th cranial nerves, and bilateral cortical areas and thickening of the adjacent leptomeningeal structures (Figures 1(c)–1(e)). No abnormalities were observed in the right medial rectus muscle or the lacrimal gland during the initial orbital MRI (Figure 1(b)). No abnormalities were found in the cranial MR angiography. Laboratory tests for thyroid function, erythrocyte sedimentation rates and C-reactive proteins were normal. Tests for thyroid antibodies, rheumatoid factors, antinuclear antibodies, antidouble-stranded DNA, anti-SSA, anti-SSB, anti-Jo-1, anti-Scl-70, anti-PM-Scl, anti-SM/RNP, anti-cardiolipin IgM-IgG, anti-phosphatidylserine IgM-IgG, p-ANCA/MPO, and c-ANCA/PR3 were also negative. Sugar, protein, and sodium levels in the cerebrospinal fluid (CSF) were normal. No cells were observed during the direct examination of CSF. PCR for Herpes Simplex Virus,* Cytomegalovirus*,* Brucella*, and* Mycobacterium* were negative. Serological tests for Lyme, Brucellosis, HIV, and Syphilis were negative. No bacteria or fungi were observed after gram staining of the CSF. No acid-resistant bacilli were observed in the CSF or sputum after Ehrlich-Ziehl-Neelsen staining. There were no bacterial, mycobacterial, or other fungal cultures. Following the prediagnosis of neurosarcoidosis, the serum angiotensin converting enzyme (ACE) level was 50 U/L (normal is 8–52 U/L) and the CSF IgG index was 0.2 (normal is 0.2–0.5). Type 3 oligoclonal bands were observed in the CSF and the CSF ACE level was elevated to 6.2 U/L (normal < 3 U/L). The patient, who refused bronchoscopy and brain biopsy, was diagnosed with probable neurosarcoidosis according to the Zajicek criteria. The patient was discharged after starting treatment with 1 mg/kg/day prednisone.

## 3. Conclusions

Involvement of the central nervous system is rarely reported in the literature. When central nervous system involvement is detected, it commonly presents as cranial neuropathy, aseptic meningitis, pituitary dysfunction, or cognitive impairment. Ocular involvement typically presents as uveitis and rarely as orbital involvement, affecting the lacrimal gland and extraocular muscles [4, 5].

The etiopathogenesis of sarcoidosis is not fully understood. Histopathologic studies show chronic inflammation, noncaseating granulomas, and progressive fibrosis, which is thought to be related to increased cellular responses. Granulomas are not specific for sarcoidosis and may be seen in connective tissue diseases and during infectious processes [7]. In histopathologic studies of intracranial neurosarcoidosis, leptomeningeal involvement is common and brain parenchymal involvement occurs via the adjacent perivascular spaces (Virchow–Robin spaces). Leptomeningeal involvement is more frequently observed in the cranium base. Microscopic stenoses and occlusions in the lumen are present due to epithelioid cell infiltration of arterial walls [8–10]. MRI studies have shown typical diffuse or nodular leptomeningeal involvement that, depending on the extent of granulomatous inflammation, will affect adjacent parenchymal tissues. In T2A sequences, the gadolinium-enhanced signal increases in cranial nerves, dura mater, and periventricular tissue. Sarcoidosis lesions display gadolinium enhancement and appear as nonspecific iso- or hypointense lesions in T1A sequences and hypo- or hyperintense lesions in T2A sequences [11–13].

Laboratory studies have detected lymphocytic pleocytosis, increased synthesis of protein and immunoglobulin, positive oligoclonal bands and, depending on CNS involvement, increased levels of ACE in the CSF. Studies have reported that high levels of ACE in the CSF are indicative of central nervous system involvement in 93-94% of cases [14, 15].

Isolated sarcoidosis without systemic involvement is rare and diagnosing these patients is difficult. Zajicek et al. determined diagnostic criteria for the differential diagnosis of CNS involvement. Steroids or other immunosuppressive agents may be required for treatment. In some studies, neurologic impairments are related to poor prognosis and mortality is reported to be 7% during the first five years [15, 16].

In conclusion, clinical presentations with isolated orbital or CNS involvement are rare. Orbital involvement is more common and may present as orbital masses, dacryocystitis, and, very rarely, extraocular muscle involvement. Unless adequately treated, it can spread to the central nervous system and cause morbidity.

## References

[1] Valeyre D., Prasse A., Nunes H., Uzunhan Y., Brillet P.-Y., Müller-Quernheim J. (2014). Sarcoidosis. *The Lancet*.

[2] Iwai K., Tachibana T., Takemura T., Matsui Y., Kitaichi M., Kawabata Y. (1993). Pathological studies on sarcoidosis autopsy. I. Epidemiological features of 320 cases in Japan. *Acta Pathologica Japonica*.

[3] Ferriby D., de Seze J., Stojkovic T. (2001). Long-term follow-up of neurosarcoidosis. *Neurology*.

[4] Pawate S., Moses H., Sriram S. (2009). Presentations and outcomes of neurosarcoidosis: a study of 54 cases. *QJM: An International Journal of Medicine*.

[5] Rothova A. (2000). Ocular involvement in sarcoidosis. *British Journal of Ophthalmology*.

[6] Mavrikakis I., Rootman J. (2007). Diverse clinical presentations of orbital sarcoid. *The American Journal of Ophthalmology*.

[7] Rosen Y. (2007). Pathology of sarcoidosis. *Seminars in Respiratory and Critical Care Medicine*.

[8] Vinas F. C., Rengachary S. (2001). Diagnosis and management of neurosarcoidosis. *Journal of Clinical Neuroscience*.

[9] Nozaki K., Judson M. A. (2012). Neurosarcoidosis: clinical manifestations, diagnosis and treatment. *Presse Medicale*.

[10] Navi B. B., DeAngelis L. M. (2009). Sarcoidosis presentiing as brainstem ischemic stroke. *Neurology*.

[11] Nowak D. A., Widenka D. C. (2001). Neurosarcoidosis: a review of its intracranial manifestation. *Journal of Neurology*.

[12] Shah R., Roberson G. H., Curé J. K. (2009). Correlation of MR imaging findings and clinical manifestations in neurosarcoidosis. *American Journal of Neuroradiology*.

[13] Christoforidis G. A., Spicklcr E. M., Recio M. V., Mehta B. M. (1999). MR of CNS sarcoidosis: correlation of imaging features to clinical symptoms and response to treatment. *The American Journal of Neuroradiology*.

[14] Khoury J., Wellik K. E., Demaerschalk B. M., Wingerchuk D. M. (2009). Cerebrospinal fluid angiotensin-converting enzyme for diagnosis of central nervous system sarcoidosis. *Neurologist*.

[15] Zajicek J. P., Scolding N. J., Foster O. (1999). Central nervous system sarcoidosis-diagnosis and management. *QJM—Monthly Journal of the Association of Physicians*.

[16] Lazar C. A., Culver D. A. (2010). Treatment of sarcoidosis. *Seminars in Respiratory and Critical Care Medicine*.

